# Reply to Malik et al. How to Define High-Flow Arteriovenous Fistula? Comment on “Kim et al. Hemodynamic Adaptation and Cardiac Effects of High-Flow Arteriovenous Access in Hemodialysis Patients: A Prospective Study. *J. Clin. Med.* 2025, *14*, 4556”

**DOI:** 10.3390/jcm15010115

**Published:** 2025-12-24

**Authors:** Yaeni Kim, Ji-hye Kim, Juyeon Woo, Oknan Choi, Mina Lee, Sunryoung Choi

**Affiliations:** 1 Department of Internal Medicine, Transplantation Research Center, College of Medicine, The Catholic University of Korea, Seoul 06591, Republic of Korea; yaeni@catholic.ac.kr; 2 Division of Nephrology, Department of Internal Medicine, Dialysis Access Center, Sahmyook Medical Center, 82 Mangu-ro, Dongdaemun-gu, Seoul 02500, Republic of Korea; 3 Department of Thoracic and Cardiovascular Surgery, Dialysis Access Center, Sahmyook Medical Center, 82 Mangu-ro, Dongdaemun-gu, Seoul 02500, Republic of Korea

## 1. Introduction

The comment raised by Malik et al. [1] acknowledges the core finding of the manuscript by Kim et al., specifically the paradoxical observation that hemodialysis (HD) patients identified as having a high vascular access blood flow to cardiac output (Qa/CO) ratio (>0.3) exhibited a significantly “lower” cardiac output (CO) at baseline compared to those with a normal Qa/CO ratio.

## 2. Explanation of the Paradoxical Finding (Lower CO in High Qa/CO Group)

Malik et al. noted that the general physiological intuition suggests that arteriovenous fistula (AVF) creation should lead to decreased systemic vascular resistance (SVR) and an increase in CO [1]. However, we found the opposite at baseline: the high Qa/CO group had significantly lower CO (4.82 ± 1.25 L/min vs. 5.77 ± 1.58 L/min, *p* = 0.005) and effective cardiac output (COef) (2.84 ± 0.95 L/min vs. 4.86 ± 1.54 L/min, *p* < 0.001) compared to the normal Qa/CO group. This occurred despite the high Qa/CO group having substantially higher Qa (1979.6 ± 510.5 mL/min vs. 930.4 ± 342.9 mL/min, *p* < 0.001). The core explanation is that the high Qa/CO ratio indicates that a disproportionately large fraction of the total cardiac output is being shunted through the AVF, compromising systemic perfusion, which is represented by a low effective CO (COef). We noted that the total hemodynamic burden, calculated as CO + Qa, remained statistically similar between the high Qa/CO group (6.80 ± 1.66 L/min) and the normal Qa/CO group (6.72 ± 1.72 L/min, *p* = 0.825). This suggests that while the “total” volume the heart was dealing with was similar, the “distribution” differed significantly, with the high Qa/CO group suffering from compromised systemic perfusion. While the creation of an AVF typically reduces SVR, the body may attempt to compensate for the excessive AVF flow (Qa) by increasing SVR, which supports the findings of Malik et al.’s analysis. In our study, the high Qa/CO group had a significantly higher pre-dialysis diastolic blood pressure (DBP) (76.8 ± 15.1 mmHg vs. 66.7 ± 14.4 mmHg, *p* = 0.002). Increases in DBP, along with decreases in the brachial artery resistive index (RI), were factors associated with a high Qa/CO ratio. DBP supports the hypothesis of a “rise in afterload due to systemic vasoconstriction”. Malik et al.’s analysis of their own data confirmed that patients with a higher Qa/CO ratio had a higher estimated SVR (26.1 Wood units vs. 17.7 Wood units, *p* < 0.001) and lower effective CO (3.7 L/min vs. 5.0 L/min, *p* < 0.001). They estimated a similar difference when calculating SVR differences based on our data. This increased SVR is interpreted as limiting the adequate increase in CO, resulting in lower tissue/organ perfusion (lower COef). We conclude that this finding points toward a clinical scenario where low COef in high-flow patients suggests “impaired cardiac adaptation”. These patients, who are identified by a high Qa/CO ratio but have a low CO, may represent a group suffering from insufficient CO increase.

## 3. Addressing Malik et al.’s Specific Questions

### 3.1. Offering Ideas Regarding the Definition of a High-Flow AVF

Malik et al. note that three definitions are generally used (Qa/CO ratio, Qa volume, or presence of HOHF). Our work offers data suggesting that functional and vascular parameters should be integrated into risk stratification: We identified that upper arm access, high pre-dialysis DBP, low RI, and low COef were independent risk factors for a high Qa/CO ratio. We also identified specific Qa (>1385 mL/min) and brachial artery flow (>1404.5 mL/min) cutoff values that demonstrated high diagnostic efficacy for identifying patients in the high Qa/CO (>0.3) group. Based on the findings, we emphasize that the persistently “low COef” in high-flow patients should serve as an early indicator of impaired cardiac adaptation, and surrogate markers of SVR, such as DBP and RI, should be considered alongside COef for risk stratification. This suggests moving beyond just volume criteria (Qa) or ratio criteria (Qa/CO) toward a functional definition focused on the compromise of systemic perfusion (low COef).

### 3.2. Why the Sum of Total CO and Qa Was Presented

We presented the value of CO + Qa. This measurement was included to demonstrate that the “total hemodynamic burden on the heart” was similar between the high Qa/CO group and the normal Qa/CO group at baseline (6.80 ± 1.66 L/min vs. 6.72 ± 1.72 L/min). By demonstrating similar total burden, we would like to highlight that the key problem in the high Qa/CO group was the “distribution of blood flow”, where excessive shunting (high Qa) reduced the volume available for systemic circulation (low COef and low CO).

## 4. Insights from One-Year Follow-Up (Adaptive Changes)

Our prospective data collection addresses the ongoing process of adaptation mentioned by Malik et al.: Over one year, the high Qa/CO group showed significant increases in CO (4.82 to 6.16 L/min, *p* = 0.007), CI, and COef (2.84 to 4.40 L/min, *p* = 0.001). Simultaneously, their Qa significantly decreased (1979.6 mL/min to 1696.1 mL/min, *p* < 0.001). The Qa/CO ratio also significantly decreased (0.42 to 0.30, *p* < 0.001). This improvement in CO, CI, and COef suggests “gradual hemodynamic adaptation” where the cardiovascular system compensates for the high-flow AVF. The heart may have redistributed more blood toward systemic circulation, resulting in higher effective perfusion. These adaptive changes are crucial because they suggest that the high Qa/CO patients who initially exhibit low CO/COef are, over time, attempting to compensate. We conclude that high Qa/CO ratios increase the risk of overt HOHF due to cardiac strain. The compensatory increase in COef may explain why many high-flow patients remain asymptomatic. However, patients who fail to exhibit this increase in COef despite decreasing Qa may be at higher risk for progressive cardiac dysfunction. In summary, the manuscript confirms the existence of a high Qa/CO phenotype characterized by paradoxically low cardiac output, driven by compromised systemic perfusion and increased DBP/SVR, and further reveals that this group undergoes significant cardiovascular adaptation over one year. This supports Malik et al.’s observation regarding different phenotypes and emphasizes the clinical importance of monitoring COef and SVR surrogates alongside Qa/CO to assess cardiac health and adaptation. We would also like to clarify the following regarding patient selection and cohort size. The final one-year analysis presented in Table 6 was performed on 120 patients who completed the full follow-up period and had complete echocardiographic and Qa data. This cohort represents the population in which long-term adaptive changes could be reliably assessed. Importantly, the reduction from 142 to 120 patients was due solely to loss to follow-up or transfer to other institutions for personal reasons. No patients were excluded—or lost to follow-up—because of clinical deterioration, worsening of pre-existing conditions, or heart failure exacerbation. Thus, the risk of survivorship bias is minimized, as dropout was administrative rather than clinically driven. The concern that the final cohort selectively retained only “compensators” is therefore substantially reduced. However, as acknowledged in the manuscript’s limitations, determining whether “non-compensators” ultimately experience cardiac deterioration would require a longer observation period.
